# Immunogenicity of bivalent versus monovalent mRNA booster vaccination among adult paramedics in Canada who had received three prior mRNA wild-type doses

**DOI:** 10.1099/acmi.0.000791.v3

**Published:** 2025-01-13

**Authors:** Michael Asamoah-Boaheng, David M. Goldfarb, Iryna Kayda, Justin Yap, Tracy Kirkham, Mohammad Ehsanul Karim, Paul Demers, Jeffrey M. Copp, Brian Grunau

**Affiliations:** 1 Department of Emergency Medicine, University of British Columbia, Vancouver, British Columbia, Canada; 2 Centre for Advancing Health Outcomes, St. Paul’s Hospital, Vancouver, British Columbia, Canada; 3 Faculty of Medicine, University of British Columbia, Vancouver, British Columbia, Canada; 4 British Columbia Resuscitation Research Collaborative, Vancouver, British Columbia, Canada; 5 Department of Pathology and Laboratory Medicine, University of British Columbia, Vancouver, British Columbia, Canada; 6 Faculty of Science, University of British Columbia, Vancouver, British Columbia, Canada; 7 Occupational Cancer Research Centre, Ontario Health, Toronto, Ontario, Canada; 8 Dalla Lana School of Public Health, University of Toronto, Toronto, Ontario, Canada; 9 School of Population and Public Health, University of British Columbia, Vancouver, British Columbia, Canada; 10 British Columbia Emergency Health Services, British Columbia, Canada

**Keywords:** BA.4/5 Omicron subvariant, bivalent mRNA vaccines, immunogenicity, mRNA COVID-19 vaccines, SARS-CoV-2 Omicron infection

## Abstract

**Introduction.** Comparative immunogenicity from different mRNA booster vaccines (directed at WT, BA.1 or BA.4/5 antigens) remains unclear.

**Methods.** We included blood samples from adult paramedics who received three mRNA WT-directed vaccines plus a fourth dose of the following: (1) WT monovalent, (2) Moderna BA.1-WT bivalent or (3) Pfizer BA.4/5 WT bivalent vaccine. The primary outcome was angiotensin-converting enzyme 2 (ACE2) inhibition to BA.4/5 antigen. We used optimal pair matching (using age, sex-at-birth, preceding SARS-CoV-2 infection and fourth vaccine-to-blood collection interval) to create balanced groups to individually compare each vaccine type to each other vaccine (overall, within subgroups defined by SARS-CoV-2 infection and after combining BA.1 and BA.4/5 cases). We compared outcomes with the Wilcoxon matched-pairs signed rank test.

**Results.** Overall, 158 paramedics (mean age 45 years) were included. ACE2 inhibition was higher for BA.1 compared to WT (*P*=0.002); however, no difference was detected between BA.4/5 vs. WT or BA.1 vs. BA.4/5. Among cases with preceding SARS-CoV-2, there were no detected between-group differences. Among cases without preceding SARS-CoV-2, the only detected difference was BA.1>WT (*P*=0.003). BA.1 and BA.4/5 cases combined had higher ACE2 inhibition than WT (*P*=0.003).

**Conclusion.** Omicron-directed vaccines appear to improve Omicron-specific immunogenicity; however, this appears limited to SARS-CoV-2-naive individuals.

## Data Summary

The COVID-19 Occupational Risk, Seroprevalence, and Immunity among Paramedics in Canada (CORSIP) dataset used in this study is not publicly accessible due to the confidential nature of the data, which includes blood sample data from study participants. However, it can be accessed online at https://portal.citf.mcgill.ca/ upon request.

## Introduction

The emergence of SARS-CoV-2 variants, including the Omicron sublineages, has led to questions regarding optimal ongoing vaccination strategies. Recent studies have documented reduced vaccine effectiveness of two-dose mRNA vaccination (including original Wuhan Hu 1 platform mRNA-1273 and BNT162b2) against Omicron BA.1 infection compared with earlier variants [1]; immunogenicity appears to improve after a third dose but subsequently wanes quickly thereafter [1,6]. While vaccination with three to four of the original mRNA vaccines remains highly effective against hospitalization for all variants, effectiveness 14–30 days post-fourth dose against COVID-19 is only moderate for the BA.2, BA.2.12.1 and BA.4 strains, but low against BA.5, with benefit appearing to disappear after 90 days [3].

To align vaccines with the current SARS-CoV-2 variants, vaccines directed at Omicron strains were developed [7,8]. The approved bivalent mRNA booster formulations contain both the Wuhan Hu-1 (WT) SARS-CoV-2 and Omicron (B.1.1.529) variant spike sequences. However, few studies have compared the effectiveness of bivalent booster doses against the WT mRNA monovalent vaccines. Further, the comparative immunogenicity conveyed from different mRNA booster vaccines (specifically WT vaccine vs. Moderna BA.1 vs. Pfizer BA4/5) among previously vaccinated individuals with or without preceding SARS-CoV-2 infection remains unclear.

For these reasons, we sought to compare the immunogenicity between those who received WT, BA.1 or BA.4/5 booster vaccines among adult paramedics in Canada who had previously received three WT mRNA vaccines.

## Methods

### Study design and data source

We used blood sample test results and questionnaire data from adult paramedics participating in the COVID-19 Occupational Risk, Seroprevalence, and Immunity among Paramedics in Canada (CORSIP) study. CORSIP is an observational cohort study that recruited adult paramedics to investigate COVID-19 occupational risk and SARS-CoV-2 seroprevalence among paramedics in Canada. Recruitment of participants started on 21 January 2021 and ended on 11 February 2023. The CORSIP study received approvals from the University of British Columbia (H20-03620) and the University of Toronto Health Research Ethics Boards (40435). The paramedics provided written consent upon enrolment through an internet-based portal. The study included paramedics from five provinces in western and central Canada, including Alberta, British Columbia, Manitoba, Ontario and Saskatchewan. Participants provided questionnaire responses regarding demographic characteristics (age, sex at birth and race/ethnicity), SARS-CoV-2 infections and symptoms, vaccination history (including dates and vaccination types) and medical history through an online portal, in addition to providing longitudinal blood samples every 6 months for serological testing [9].

### Study population

CORSIP participants were included in these analyses if they had received four mRNA vaccine doses prior to providing a blood sample, with the first three doses being a WT-based mRNA vaccine, and the fourth dose being either of the following: (1) a Moderna BA.1 bivalent vaccine, directed at BA.1 and WT strains (hereafter referred to as ‘BA.1 vaccine’); (2) a Pfizer BA.4/5 bivalent vaccine, directed at BA.4/5 and WT strains (hereafter referred to as ‘BA.4/5 vaccine’); or (3) a WT mRNA monovalent vaccine, directed only at the WT strain (hereafter referred to as ‘WT vaccine’). Participants who had received any non-mRNA vaccines were excluded. The study cohort included both those who had and had not previously been infected with SARS-CoV-2. SARS-CoV-2 infection was defined as (1) a participant-reported positive SARS-CoV-2 nucleic acid amplification test or rapid antigen test result or (2) the presence of anti-nucleocapsid antibodies in the blood sample (Roche, IND, USA).

Given that we aimed to investigate the impact of the fourth vaccine (after three WT-based monovalent mRNA vaccines), we categorized cases based on the type of fourth vaccine: BA.1 vaccine, BA.4/5 vaccine or WT vaccine. We chose samples (as described below) from eligible participants to create matched groups for three separate comparisons to compare each vaccine type to each other vaccine type.

### Serological testing

We tested all blood samples with (1) the V-PLEX SARS-CoV-2 Panel 28 ACE2 Kit (Meso Scale Discovery, MD, USA) to measure the per cent inhibition of angiotensin-converting enzyme 2 (ACE2) binding to spike protein of Omicron (B.1.617.2) BA.4/BA.5 sub-lineages and (2) the Elecsys Anti-SARS-CoV-2 Nucleocapsid assay (Roche IND, USA) to identify the presence of anti-nucleocapsid protein antibodies, indicative of preceding SARS-CoV-2 infection. This Elecsys assay has previously been shown to have high sensitivity for detecting preceding SARS-CoV-2 infections [10].

### Outcome variable

The primary outcome was ACE2 per cent (%) inhibition to the BA.4/5 Omicron antigen.

### Statistical analysis

We described continuous variables with a mean (and sd) or median [with interquartile range (IQR)] and represented categorical variables with counts (with percentages). We used GraphPad Prism version 9.5.0 (GraphPad Software, San Diego, CA) and SAS version 9.4 for data analyses.

We performed a 1 : 1 matching to select the multiple pairs from the three vaccine types using the optimal pair matching method [11,12]. For the first main comparison, comparing BA.1 vaccine vs. WT vaccine, we used the optimal pair matching method to create two balanced groups for comparison, matching based on participant age, sex at birth, preceding SARS-CoV-2 infection and the number of days from the fourth dose to blood collection. We repeated this process for the second main comparison of BA.1 vaccine vs. BA.4/5 vaccine and again for the third main comparison of BA.4/5 vaccine vs. WT vaccine.

In addition, we performed several sensitivity analyses. First, we repeated the comparisons but only included samples from participants who had evidence of preceding SARS-CoV-2 infections. Second, we repeated the comparisons including only samples from participants who did not have preceding SARS-CoV-2 infections. Third, we combined all the BA.4/5 and BA.1 vaccine cases and compared them to the WT vaccine cases (overall and within subgroups dichotomized by preceding SARS-CoV-2 infection).

We diagrammed ACE2 inhibition to BA.4/5 for the paired comparisons using scatter plots and used the Wilcoxon matched-pairs signed rank test to test the differences between the median ACE2 inhibitions to BA.4/5 of the groups. Further, we used the Related-Samples Hodges–Lehman test to estimate the CI of the differences in medians. In addition, we used the Bonferroni correction test to adjust *P*-values obtained from the Wilcoxon matched-pairs signed rank test in order to account for multiple comparisons of outcomes.

## Results

Overall, the study included a total of 158 paramedics, with a mean participant age of 45 years. Characteristics of participants included in the three different comparisons are shown in Tables 1, S1 and S2 (available in the online version of this article). Comparison group characteristics, and the interval between the fourth vaccine to blood collection, were similar.

**Table 1.** Participant characteristics stratified by matched pairs of vaccine doses (including those with or without preceding COVID-19)

	Comparison 1*		Comparison 2*		Comparison 3*	
**Variables**						
	Moderna BA.1	WT	Moderna BA.1	Pfizer BA.4/5	Pfizer BA.4/5	WT
	*n*=61	*n*=61	*n*=34	*n*=34	*n*=34	*n*=34
***Matched variables***						
Age in years, mean (sd)	45 (11)	45 (10)	45 (11)	43 (10)	43 (10)	44 (11)
Sex (at birth), n (%)						
Female	22 (36)	23 (38)	13 (38)	15 (44)	15 (44)	16 (47)
Male	39 (64)	38 (62)	21 (62)	19 (56)	19 (56)	18 (53)
COVID-19 diagnosis, total [n (%)]	37 (61)	32 (53)	22 (65)	24 (71)	24 (71)	21 (62)
Pre-Omicron infections†	2 (5)	2 (6)	2 (9)	2 (8)	2 (8)	2 (10)
Omicron infections†	35 (95)	30 (94)	20 (91)	22 (92)	22 (92)	19 (90)
V4-to-BC (days), median (IQR)	81 (48, 119)	72 (41, 123)	60 (43,79)	54 (42, 75)	54 (42, 75)	47 (37, 85)
***Other variables***						
*Race/ethnicity*, n (%)						
Racialized	6 (10)	5 (8)	4 (12)	5 (15)	5 (15)	32 (94)
White	55 (90)	56 (92)	30 (88)	29 (85)	29 (85)	2 (6)
Tobacco use, n (%)	3 (5)	4 (7)	2 (6)	1 (3)	1 (3)	3 (9)
Influenza vaccination, n (%)	54 (89)	54 (89)	30 (88)	30 (88)	30 (88)	31 (91)
*Medical history, n (%*)						
Hypertension	5 (8)	6 (10)	4 (12)	5 (15)	5 (15)	5 (15)
Diabetes	2 (3)	5 (8)	2 (6)	–	1 (2.9)	–
Asthma	10 (16)	5 (8)	4 (12)	1 (3)	1 (2.9)	2 (6)
Chronic lung disease	–	–	–	1 (3)	1 (2.9)	1 (3)
Cancer	2 (3)	1 (2)	1 (3)	4 (12)	4 (12)	1 (3)

WT: wild-type; sd: standard deviation; IQR: interquartile range; V4-to-BC: days from vaccine 4 to the blood collection date.

**No significant differences between vaccine groups, within comparison, were observed for characteristics.

**†Pre-Omicron infection was defined as SARS-CoV-2 infections that occur before 26 November 26, 2021; and Omicron infections were defined as infections that occurred after 26 December 26, 2021 (A[as majority (95%) of Omicron infections occurred in Canada after 26 December 26, 2021)].

**Fig. 1.**
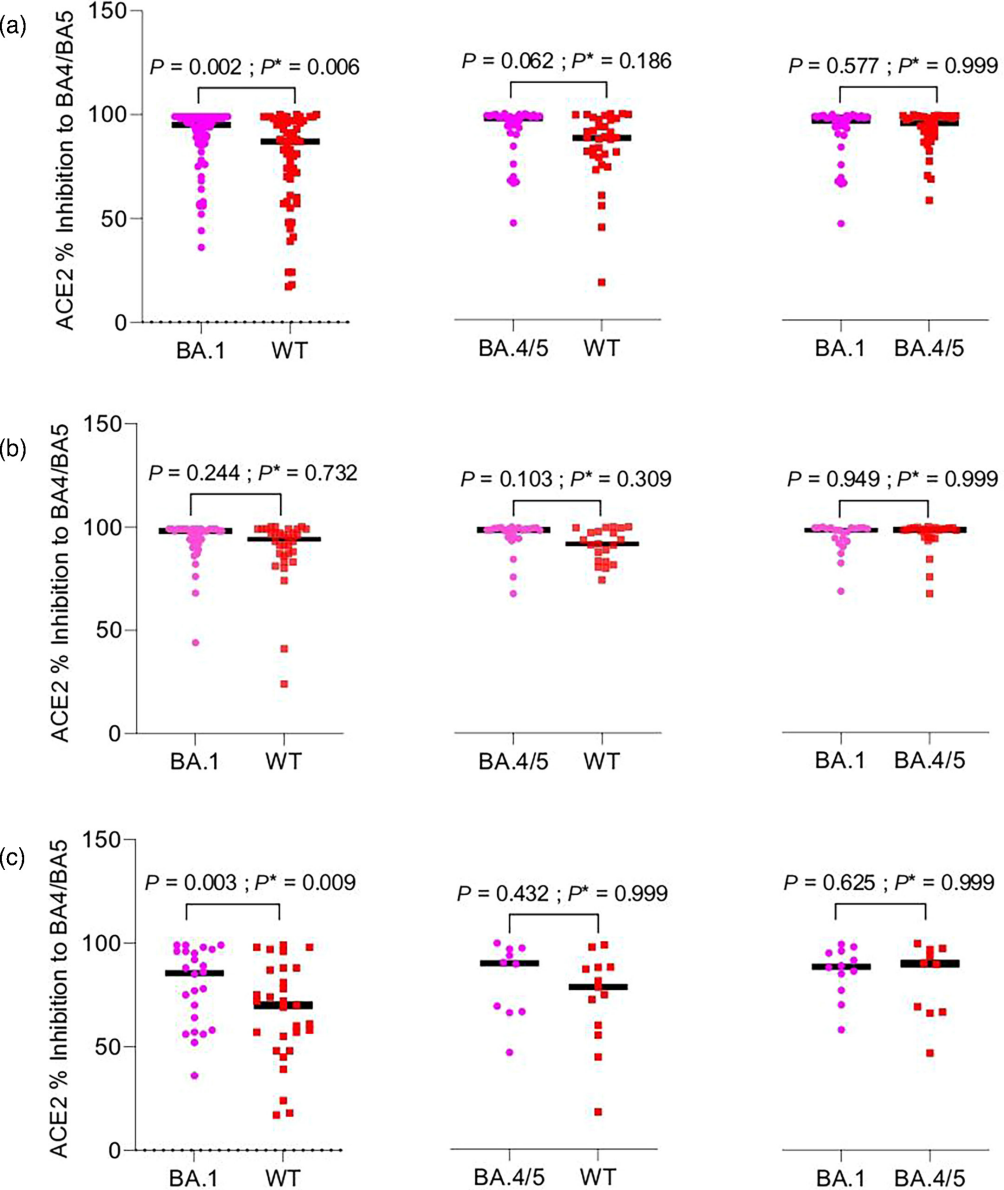
Comparing immunogenicity of bivalent vaccine doses with monovalent vaccine doses among all participants (**a**), among participants with previous SARS-CoV-2 infections (**b**) and among participants with no previous SARS-CoV-2 infection (**c**). The solid horizontal line (in the plots) denotes the median ACE2 inhibitions. Outcomes were compared with the Wilcoxon matched-pairs signed rank test, with the original *P* value (*P*) and Bonferroni-corrected *P*-value (P*) as shown.

Fig. 1(a) shows the results of the main comparisons, including all samples (from both individuals with and without preceding SARS-CoV-2 infections). We observed a significantly higher per cent inhibition to BA.4/5 among participants who received a fourth BA.1 vaccine compared to those who received a fourth WT vaccine [median difference (Mdn): 9.0, 95% CI: 3.8, 14.4, P* = 0.006; Fig. 1a (left)]. We did not observe a statistically significant difference in the second main comparison between the BA.4/5 vaccine vs. WT vaccine [Mdn: 7.1, 95 % CI: −0.52, 13.7, P* = 0.186; Fig. 1a (middle)], nor in the third main comparison, between the BA.1 vaccine vs. BA.4/5 vaccine [Mdn=0.56, 95 % CI: −3.0, 3.9, P* = 0.999; Fig. 1a (right)].

Fig. 1(b) shows the results of the three comparisons when including only samples from individuals with preceding SARS-CoV-2 infections. We did not observe a statistically significant difference in per cent inhibitions for any comparison.

Fig. 1(c) shows the results of the three comparisons when including only samples from individuals without preceding SARS-CoV-2 infections. We observed a significantly higher per cent inhibition for the BA.1 vaccine when compared to the WT vaccine; however, we did not observe a statistically significant difference in the other two comparisons.

In the fourth analysis, samples from individuals who received either BA.1 or BA.4/5 vaccines had higher levels of ACE2 per cent inhibition to BA.4/5 compared to those who received the WT vaccine (Mdn=7.8, 95 % CI: 1.5, 16, P* = 0.009; Fig. S1A). This was consistent when comparing samples from individuals who had not previously had a SARS-CoV-2 infection (Mdn=17.7, 95 % CI: 7.9, 26; Fig. 1c); however, a difference was not seen when comparing samples from individuals who had previously had SARS-CoV-2 infections (Mdn=2.8, 95 % CI: −1.2, 8.1; Fig. S1B).

## Discussion

The results of this work provide insights into the humoral immune response to the Omicron BA.4/5 subvariant among individuals vaccinated with different types of mRNA COVID-19 boosters. A unique aspect of this study is that it occurred in the Canadian context, where both the Moderna BA.1 and Pfizer BA.4/5 vaccines were introduced, allowing for a comparison of the humoral response elicited by both of these vaccines against a more recent variant. Among individuals who had already received three prior WT monovalent mRNA vaccines, we were able to compare those who received an additional WT vaccine, a BA.1 bivalent vaccine or a BA.4/5 bivalent vaccine for their fourth dose and examined resultant immunogenicity against a BA.4/5 antigen. Overall, bivalent fourth vaccines demonstrated superior immunogenicity compared to the fourth WT vaccine among SARS-CoV-2-naïve individuals. Interestingly, our results did not suggest that the BA.4/5-directed vaccine was superior to those targeting BA.1. This suggests that both bivalent boosters may be equally effective in boosting humoral immunity against the Omicron BA.4/5 subvariant, which is in keeping with recent vaccine effectiveness data from Denmark comparing these two bivalent booster vaccines [13].

We found that neither bivalent vaccine demonstrated an advantage over WT vaccine boosting in those with evidence of prior SARS-CoV-2 infection; however, the majority of those in our cohort had evidence of infection during the Omicron era, which may help explain this finding. Although we could not detect a difference between the BA.4/5 vaccine and the WT vaccine, this may have been due to a smaller sample size compared to the BA.1 vs. WT comparison. This could also be attributable to the fact that the timing of the fourth monovalent dose to the blood draw was shorter than that of the fourth dose of BA.4/5 booster to the blood draw, thereby reducing the expected differences in immunogenicity between the two boosters. Further, the higher dose of the Moderna BA.1 booster (50 mcg) compared to the Pfizer BA.4/5 booster (30 mcg) could also help explain these findings. These results are also consistent with previous evidence demonstrating that the higher-dosed mRNA-1273 vaccine resulted in superior serological markers at 6 months compared to those who received the BNT162b2 vaccine [14].

Overall, our results suggest that the Omicron-specific bivalent booster may be more effective in boosting humoral immunity against the Omicron BA.4/5 subvariant than using all four WT monovalent vaccines. However, the effect appears to be limited to those who have never been infected with SARS-CoV-2. Additionally, our findings suggest that multiple exposures to the original WT mRNA vaccines, followed by a bivalent booster, enhance immune responses, particularly in SARS-CoV-2-naive individuals compared to fewer doses or single bivalent exposures. This observation could imply that a strategy involving sequential boosters, including a bivalent dose, may confer benefits in sustaining immunity over time.

These findings have important implications for ongoing COVID-19 vaccination strategies, especially in jurisdictions where the Omicron variant is prevalent. Further research is needed to confirm these results in other occupational groups and to investigate the long-term effectiveness of different COVID-19 booster vaccines in inducing and maintaining humoral immunity.

### Limitations

Our study might have been subjected to some limitations. This study may not be generalizable to the greater population, given that our study population included a relatively homogenous group of middle-aged adult paramedics whose immunity may differ from other groups. Other limitations include lack of randomization, use of self-reported data and small sample size, which might have influenced the findings of some comparisons between bivalent and WT boosters.

## Conclusion

Our study suggests that the Omicron-specific bivalent boosters may induce a higher humoral immune response against the Omicron BA.4/5 subvariant compared to the mRNA monovalent boosters. However, this finding appears limited to those without preceding SARS-CoV-2 infections.

## supplementary material

10.1099/acmi.0.000791.v3 Uncited Supplementary Material 1.

## Abbreviations

ACE2: angiotensin-converting enzyme 2
CORSIP: COVID-19 occupational risk, seroprevalence, and immunity among paramedics
IQR: interquartile range
Mdn: median difference
