# Supplementary material for: Immunogenicity of bivalent versus monovalent mRNA booster vaccination among adult paramedics in Canada who had received three prior mRNA wild-type doses
Source: Access Microbiol. 2025 Jan 13;7(1):000791.v3. doi: 10.1099/acmi.0.000791.v3 (PMC11728694; doi:10.1099/acmi.0.000791.v3)
Supplement: Uncited Supplementary Material 1. [file acmi-7-00791-s001.pdf]

**Supplementary Table 1:** Participants characteristics among individuals without preceding COVID-19 diagnosis

| Variables | Comparison 1* | | Comparison 2* | | Comparison 3* | |
|---|---|---|---|---|---|---|
| | Moderna BA. 1 | WT | Moderna BA.1 | Pfizer BA.4/5 | Pfizer BA 4/5 | WT |
| | n = 24 | n = 29 | n = 12 | n = 10 | n = 10 | n =13 |
| ***Matched variables*** | | | | | | |
| Age, Mean (SD) | 47 (11) | 44 (9) | 48 (10) | 40 (8) | 40 (8) | 43 (10) |
| Sex (at birth) | | | | | | |
| Female | 9 (38) | 9 (31) | 5 (42) | 4 (40) | 4 (40) | 6 (46) |
| Male | 15 (62) | 20 (69) | 7 (58) | 6 (60) | 6 (60) | 7 (54) |
| V4-to-BC (days) | 77 (53, 118) | 70 (41, 88) | 67 (45, 78) | 56 (51, 90) | 56 (51, 90) | 42 (36, 80) |
| ***Other variables*** | | | | | | |
| *Race/Ethnicity*, n (%) | | | | | | |
| Racialized | 3 (12) | 1 (3) | 3 (25) | 3 (30) | 3 (30) | 1 (8) |
| White | 21 (88) | 28 (97) | 9 (75) | 7 (70) | 7 (70) | 12 (92) |
| Tobacco use, n (%) | 3 (13) | 2 (7) | 2 (17) | 0 (0) | 0 (0) | 1 (8) |
| Influenza vaccination, n (%) | 21 (88) | 24 (83) | 11 (92) | 7 (70) | 0 (0) | 11 (85) |
| *Medical history, n (%)* | | | | | | |
| Hypertension | 1 (4.2) | 1 (3) | 0 (0) | 0 (0) | 0 (0) | 0 (0) |
| Diabetes | 0 (0) | 0 (0) | 0 (0) | 0 (0) | 0 (0) | 0 (0) |
| Asthma | 6 (25) | 4 (14) | 2 (17) | 0 (0) | 0 (0) | 1 (8) |
| Chronic Lung Disease | - | - | 0 (0) | 0 (0) | 0 (0) | 0 (0) |
| Cancer | 1 (4) | 1 (3) | 0 (0) | 2 (20) | 2 (20) | 1 (8) |

**WT:** Wild type; SD: Standard deviation, IQR: Interquartile range; V4-to-BC: Days from vaccine 4 to the last blood collection date.

*No significant differences between vaccine groups, within comparison, was observed for characteristics.

**Supplementary Table 2:** Participants characteristics among individuals with preceding COVID-19 diagnosis

| **Variables** | **Comparison 1*** | | **Comparison 2*** | | **Comparison 3*** | |
|---|---|---|---|---|---|---|
| | Moderna BA. 1 | WT | Moderna BA.1 | Pfizer BA.4/5 | Pfizer BA 4/5 | WT |
| | n = 37 | n = 32 | n = 22 | n = 24 | n = 24 | n = 21 |
| ***Matched variables*** | | | | | | |
| Age, Mean (SD) | 44 (10) | 45 (11) | 43 (11) | 44 (11) | 44 (11) | 44 (12) |
| Sex (at birth) | | | | | | |
| Female | 13 (35) | 14 (44) | 8 (36) | 11 (46) | 11 (46) | 10 (48) |
| Male | 24 (65) | 18 (56) | 14 (64) | 13 (54) | 13 (54) | 11 (52) |
| V4-to-BC (days) | 83 (47, 120) | 93 (41, 129) | 53 (42, 82) | 47 (41, 74) | 43 (36, 51) | 42 (36, 54) |
| ***Other variables*** | | | | | | |
| *Race/Ethnicity*, n (%) | | | | | | |
| Racialized | 3 (8) | 4 (12) | 1 (4) | 2 (8) | 2 (8) | 1 (5) |
| White | 34 (92) | 28 (88) | 21 (96) | 22 (92) | 22 (92) | 20 (95) |
| Tobacco use, n (%) | 0 (0) | 2 (6) | 0 (0) | 1 (4) | 1 (4) | 2 (10) |
| Influenza vaccination, n (%) | 4 (11) | 30 (94) | 19 (86) | 23 (96) | 23 (96) | 20 (95) |
| *Medical history, n (%)* | | | | | | |
| Hypertension | 4 (11) | 5 (16) | 4.0 (18) | 5.0 (21) | 5 (21) | 4 (19) |
| Diabetes | 2 (5) | 0 (0.0) | 2.0 (9) | 0 (0) | 0 (0) | 0 (0) |
| Asthma | 4 (11) | 1 (3) | 2 (9) | 1 (4) | 1 (4) | 1 (5) |
| Chronic Lung Disease | 0 (0) | 0 (0) | 0 (0) | 1 (4) | 1 (4) | 1 (5) |
| Cancer | 1 (3) | 0 (0) | 1 (5) | 2 (8) | 2 (8) | 0 (0) |

**WT:** Wild type; SD: Standard deviation, IQR: Interquartile range; V4-to-BC: Days from vaccine 4 to the last blood collection date

*No significant differences between vaccine groups, within comparison, was observed for characteristics.

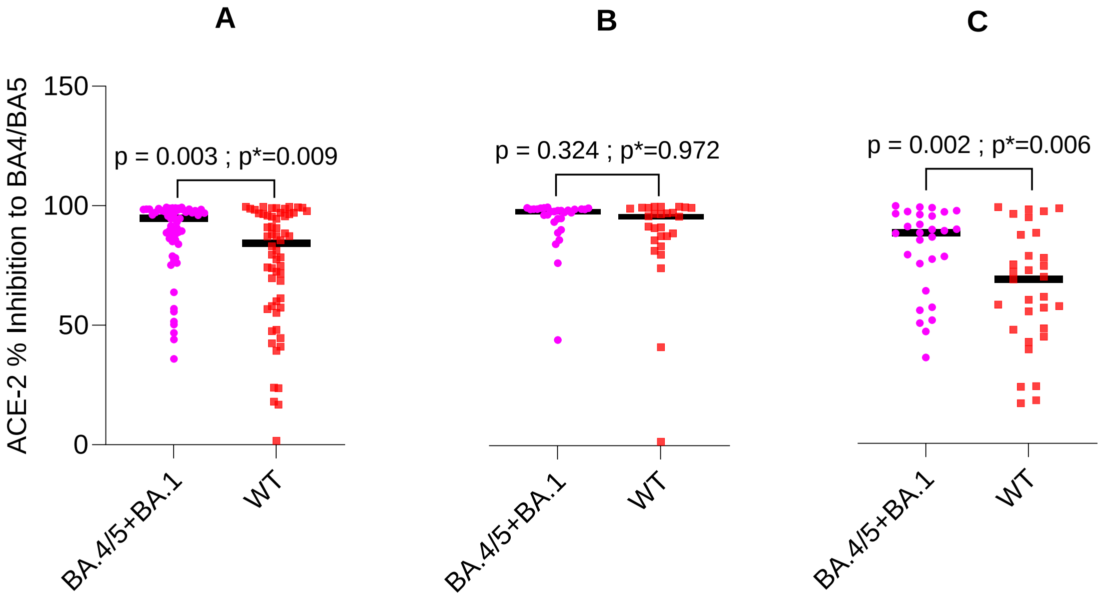


**Supplementary Figure 1**: Comparing immunogenicity of the combined bivalent booster with the fourth dose monovalent vaccine booster.

**A**: The plot compares the fourth dose of combined bivalent booster (including BA.1 or BA.4/5) versus the fourth dose of monovalent booster among paramedics with or without preceding SARS-CoV-2 infections.

**B**: The plot compares the fourth dose of combined bivalent booster (including BA.1 or BA.4/5) versus the fourth dose of monovalent booster among paramedics with preceding SARS-CoV-2 infections.

**C**: The plot compares the fourth dose of combined bivalent boosters (including BA.1 or BA.4/5) versus the fourth dose of monovalent booster among paramedics without preceding SARS-CoV-2 infections.

The solid line denotes the median ACE-2 inhibitions. Outcomes were compared with the Wilcoxon matched pairs signed rank test, with the original p value (p) and Bonferroni corrected p-value (p*) as shown.
